# *XRCC1* codon 399Gln polymorphism is associated with radiotherapy-induced acute dermatitis and mucositis in nasopharyngeal carcinoma patients

**DOI:** 10.1186/1748-717X-8-31

**Published:** 2013-02-01

**Authors:** Haijun Li, Yanjie You, Canfeng Lin, Mingzhang Zheng, Chaoqun Hong, Jiongyu Chen, Derui Li, William W Au, Zhijian Chen

**Affiliations:** 1 Department of Radiation Oncology, Cancer Hospital of Shantou University Medical College, 7 Raoping Road, Shantou, Guangdong, 515041, China; 2 Central laboratory, Cancer Hospital of Shantou University Medical College, 7 Raoping Road, Shantou, Guangdong, 515041, China; 3 Cancer research center, Shantou University Medical College, 7 Raoping Road, Shantou, Guangdong, 515041, China; 4 Faculty of Preventive Medicine, Shantou University Medical College, 22 Xinling Road, Shantou, Guangdong, 515041, China

**Keywords:** Single nucleotide polymorphisms (SNPs), X-ray cross-complementing group 1 (XRCC1), Nasopharyngeal carcinoma (NPC), Radiotherapy, Acute skin reactions, Acute mucosa reactions

## Abstract

**Background:**

To evaluate the association between single nucleotide polymorphisms (SNPs) at the 194 and 399 codons of *XRCC1,* and the risk of severe acute skin and oral mucosa reactions in nasopharyngeal carcinoma patients in China.

**Methods:**

114 patients with nasopharyngeal carcinoma were sequentially recruited in this study. Heparinized peripheral blood samples were taken for SNPs analysis before the start of radiation treatment. SNPs in *XRCC1* (*194Arg/Trp* and *399Arg/Gln*) gene were analyzed by polymerase chain reaction-restriction fragment length polymorphism (PCR-RFLP). Dermatitis at upper neck and oral mucositis were clinically recorded according to the Common Terminology Criteria for Adverse Events v.3.0.

**Results:**

The variant allele frequencies were 0.289 for *XRCC1 194Trp* and 0.263 for *XRCC1 399Gln*. Of the 114 patients, 24 experienced grade 3 acute dermatitis and 48 had grade 3 acute mucositis. The *XRCC1 399Arg/Gln* was significantly associated with the development of grade 3 dermatitis (Odds Ratio, 2.65; 95% CI, 1.04–6.73; *p* = 0.037, *χ2* = 4.357). In addition, it was also associated with higher incidence of grade 3 mucositis with a borderline statistical significance (Odds Ratio, 2.11; 95% CI, 0.951–4.66; *p* = 0.065, *χ2* = 3.411). The relationship between XRCC1 *194Arg/Trp* and acute dermatitis, and mucositis was not found.

**Conclusions:**

Our investigation shows, for the first time, that patients with the *XRCC1 399Arg/Gln* genotype were more likely to experience severe acute dermatitis and oral mucositis. With further validation, the information can be used to determine personalized radiotherapy strategy.

## Background

Nasopharyngeal carcinoma (NPC) is prevalent in certain parts of the world: Southern China, North Africa, and parts of the Mediterranean basin [1]. Radiation therapy (RT) alone or in combination with chemotherapy are the standard treatments of NPC and the overall 5 year survival rate is between 59% and 85% [2,3]. However, radiation-induced toxicity limits the use of higher doses to further improve efficacy. Balancing between treatment efficiency and toxicity of RT is always a dilemma to radiation oncologist.

The severity of RT-induced complication is associated with many factors: radiation dose, combined with chemotherapy, types of tissues, and microenvironment of involved tissues. However, similar patients who received similar or identical treatment protocols had substantially different degree of clinical side effects. Therefore, genetic component may contribute to the clinical radiosensitivity [4-6].

As RT exerts its cytotoxic effects through damage to cells, proteins, and DNA, individual variations in the repair of DNA damage may modify the response of normal tissues [7]. Polymorphisms of X-ray repair cross-complementing group 1 (*XRCC1*), a base excision repair (BER) gene, was shown to affect DNA repair activity, cell cycle progression, and sensitivity to bleomycin, a radiation-mimicking agent that induces double-strand breaks in DNA [8,9].

In NPC patients, the 399Gln variant XRCC1 gene was reported to be associated with lower grade fibrosis after radiation therapy in a small number of patients [10]. Using immunohistochemical techniques, the lack of *ERCC1* staining was associated with increased radiosensitivity [11]. On the other hand, high staining intensity of the ERCC1 protein was associated with poor loco-regional control of the carcinoma [12]. Clearly, these studies do not provide consistent or adequate information on radiosensitivity that can be used to modify therapeutic protocols. Therefore, we have conducted our study using an enhanced experimental design.

Numerous studies have examined the association of DNA repair SNPs with acute and late RT normal tissue effects. XRCC1 gene was the one frequently investigated in DNA repair gene family. The results of association between XRCC1 SNP and radio- response were in consistent [13]. From this point, we have conducted this genetic polymorphism study on relevant *XRCC1* alleles. In addition, we associated the polymorphisms with acute mucositis and dermatitis which are common side-effects from radiation therapy among NPC patients.

## Methods

### Patient characteristics and clinical parameters

Between Nov, 2009 and Dec, 2010, 114 patients with histologically confirmed NPC were recruited to this study in the Cancer Hospital, Shantou University Medical College, China. The study was approved by the local ethics committee and informed consent was obtained from all patients enrolled. Patients without systemic metastasis and receiving definite RT alone or combined with chemotherapy were eligible to the study. In the period of study, totally 380 consecutive patients were eligible to the study. Among them, 114 patients consented to the study.

Evaluation by physical examination, nasopharyngeal endoscope, Magnetic Resonance Imaging of nasopharynx and neck, chest X-ray, and abdominal ultrasonography were routinely performed for tumor staging. Skeletal scanning by single photon emission computed tomography was selected to patients with stage N2-3 disease. The overall stage distribution was 11.4% (13/114) for stages II; 40.4% (46/114) for stage III; and 48.2% (55/114) for stages IV by AJCC staging system V7 [14].

The enrolled patients consisted of 36 females and 78 males with age of 19–76 (mean 49.6, SD 11.9). One hundred and twelve patients had World Health Organization (WHO) type II or III tumor. Two had WHO type I tumor. Ninety-five patients received combined chemo-radiotherapy with cisplatin-based single or doublet scheme, and 19 received radiotherapy alone. The combination strategies were selected based on tumor stage and patient’s performance status. Three-dimensional conformal radiation therapy (3D-CRT) or intensity modulated radiotherapy (IMRT) were used for radiotherapy. In using 3D-CRT, the prescription doses were 70 Gray (Gy) in 35 fractions (Fs) (70Gy/35Fs) to primary tumor, 68Gy/34Fs to involved cervical drainage regions, and 50Gy/25Fs to uninvolved cervical drainage regions. In using IMRT, the prescription doses were 70Gy/32-33Fs to primary tumor, 66-68Gy/32-33Fs to cervical nodular lesions, 60Gy/32-33Fs to involved cervical drainage regions, and 54Gy/32-33Fs to uninvolved cervical drainage regions. All radiotherapies were delivered once daily, 5 days weekly.

### Study endpoints

Radiation-induced acute dermatitis and mucositis were the endpoints of this study. The upper neck was selected as evaluation area, considering all patients received irradiation to their upper neck. In addition, the foldless skin of upper neck might eliminate the unplanned extra dose to the folded region caused by build-up effect of beams. The acute dermatitis and mucositis were scored by the Common Terminology Criteria (CTC) for adverse events v3.0 scale [15]. The highest grade of toxicity was chosen as the reference value. It was evaluated and documented weekly from the beginning to the end of radiotherapy. All scores of dermatitis and mucositis were confirmed by the same senior consultant physician as to eliminate the observer bias.

### Calculation of radiation doses to interesting regions

The irradiation dose to the observed skin areas was generated by the method reported in the literature previously [6]. Briefly, a rim of 5mm beneath the external contour of upper neck was created and used as a surrogate for the epidermis and dermis, which extended to the edge of mandible cranially and level C6 vertebra caudally. The average dose to the rim was calculated to represent the doses received by the observed skin. The average dose to oral cavity was taken as the reference dose received by oral mucosa. In axis CT image, the region between the outer edge of gum and anterior edge of tonsillar fossa was contoured as oral cavity, which extended from the floor of the mouth to the hard palate and soft palate. All contouring and calculations were performed using the Pinnacle3® (version 8.0m) treatment planning system (Phillips, Madison, WI, US).

### Genotyping analysis

A heparinized peripheral blood sample of 1ml was taken before the start of treatments. Genomic DNA was extracted from whole blood samples using the TIANamp Extraction Kit (Tiangen, Beijing, China). The polymorphic sites in *XRCC1* (*194Arg/Trp* and *399Arg/Gln*) were analyzed by Polymerase Chain Reaction–Restriction Fragment Length Polymorphism (PCR- RFLP) using Plantinum Taq DNA polymerase (Invitrogen, Shanghai, China) for PCR and *PvuII* and *MspI* (NEB, Beijing, China) for digestion as reported in literature [8]. In addition, 10% of randomly selected samples were sequenced to verify genotyping results and 100% concordance was found.

### Statistical analysis

All analyses were performed using SPSS software, version 13.0 (SPSS, Chicago, IL). Each polymorphism was tested for deviation from Hardy-Weinberg equilibrium by comparing the observed and expected genotype frequencies using the chi-square test with 1° of freedom. A *p* value of 0.05 or less (in a two-sided test) was considered statistically significant. Patients were subdivided according to their CTC score of dermatitis and mucositis. Patients who experienced episodes of grade 3 or higher toxicity were defined as radiation sensitive (Grade 3) and those with milder toxicity were defined as radiation insensitive (Grade 1–2). The Grade 3 vs. Grade 1–2 frequencies were examined by calculating the odds ratios (OR), with 95% confidence interval (95% CI) using the chi-square test, with that of wild-type alleles as the reference.

The effect of age, gender, smoking, alcohol drinking, body mass index, tumor stage, radiotherapy technique, and radiation dose to observed tissue volumes on clinical radiosensitivity was also evaluated using the student's *t* test or the chi-square test.

## Results

### Over all acute reactions of skin and oral mucosa

Acute radiation-induced dermatitis in the upper neck and mucositis in the oral cavity were the clinical endpoints of this study. Of the 114 patients, 31 (27.2%) experienced grade 1, 59 (51.8%) grade 2, and 24 (21.1%) grade 3 skin toxicity. In addition, 24 (21.1%) had grade 1, 42 (36.8%) grade 2, and 48 (42.1%) grade 3 acute oral mucositis. There was no grade 4 or grade 5 toxicities of skin or mucosa observed during the treatment.

### Distribution of allele frequencies for XRCC1 (194Arg/Trp and 399Arg/Gln)

Overall, the variant allele frequencies were 0.289 for *XRCC1 194Trp* and 0.263 for *XRCC1 399Gln*. All the genotype distributions were in Hardy-Weinberg equilibrium (p>0.05 at the chi-square for each allele).

Because of low frequency (<10%) of the genotypes of *XRCC1* (*194 Trp/Trp*) and *XRCC1* (*399 Gln/Gln*), we did not calculate the association between each of these genotypes and acute reactions.

### Polymorphisms in XRCC1 gene and skin reaction

Table 1 shows the distribution of alleles *Arg/Trp*, *Arg/Trp* or *Trp/Trp* for *XRCC1* at codon 194. Compared with *XRCC1* 194 *Arg/Arg* (wild-type)*,* the *XRCC1 194Arg/Trp* was associated with lower incidence of grade 3 acute skin toxicity. The risk odds rate was 0.70 or less. However, the difference was not statistically significant.

**Table 1:** **Association of polymorphisms in *XRCC1 *and the risk of acute dermatitis**

	**Dermatitis**			
**Genotype**	**Grade 1–2**	**Grade 3**	**OR (95% CI)**	***p*value**
*XRCC1* (*194Arg/Trp*)				
*Arg/Arg* (wild-type)	41	14	1.00 (ref)	
*Arg/Trp*	42	10	0.697 (0.278–1.75)	0.440
*Trp/Trp*	7	0	NC	NC
*XRCC1* (*399Arg/Gln*)				
*Arg/Arg* (wild-type)	53	10	1.00 (ref)	
*Arg/Gln*	28	14	2.65 (1.04–6.73)	0.037
*Gln/Gln*	9	0	NC	NC

Abbreviations: *ref* = reference; *NC* = not calculated; *OR* = Odds ratio; *95%CI* = 95% confidence interval.

The *XRCC1 399Arg/Gln* was associated with high risk of grade 3 skin toxicity, OR = 2.65 (95% CI: 1.04–6.73), compared to the wild-type. It was statistically significant as tested by the chi-square test (*p* = 0.037, *χ2* = 4.357).

### Polymorphisms in XRCC1 gene and reaction of oral mucosa

In Table 2, the results show that the *XRCC1 194Arg/Trp* was associated with lower incidence of grade 3 acute oral mucositis, compared with the *Arg/Arg* allele. But it was not statistically significant.

**Table 2:** **Association of polymorphisms in *XRCC1 *and the risk of acute oral mucositis**

	**Mucositis**			
**Genotype**	**Grade 1–2**	**Grade 3**	**OR (95% CI)**	***p*value**
*XRCC1* (*194Arg/Trp*)				
*Arg/Arg* (wild-type)	31	24	1.00 (ref)	
*Arg/Trp*	33	19	0.744 (0.342–1.62)	0.454
*Trp/Trp*	2	5	NC	NC
*XRCC1* (*399Arg/Gln*)				
*Arg/Arg* (wild-type)	40	23	1.00 (ref)	
*Arg/Gln*	19	23	2.11 (0.951–4.66)	0.065
*Gln/Gln*	7	2	NC	NC

Abbreviations: *ref* = reference; *NC* = not calculated; *OR* = Odds ratio; *CI* = confidence interval.

Compared to the wild type, *XRCC1 399Arg/Gln* was associated with higher incidence of grade 3 oral mucosa toxicity, OR = 2.11 (95% CI: 0.951-4.66). The difference was borderline statistically significant, as tested by chi-square (*p* = 0.065, *χ2* = 3.411).

### Clinical factors and acute reactions

The relationship between acute reaction of skin and mucus, and factors of age, gender, smoking, alcohol drinking, body mass index, tumor stage, and chemotherapy, radiotherapy technology, and radiation dose to observed tissue volumes were also evaluated with univariate analyses. No linkage between the observed endpoint and the above mentioned factors were found (details are shown in Table 3).

**Table 3:** Association of clinical factors and the risk of acute skin and oral mucosa reactions

	**Dermatitis**			**Mucositis**		
	**Grade 1–2**	**Grade 3**	***p*value**	**Grade 1–2**	**Grade 3**	***p*value**
Age (year)						
Mean±SD	49.3±12.3	50.8±10.2	0.587	48.2±12.5	51.5±10.7	0.144
Gender						
Male	61	17	0.775	48	30	0.246
Female	29	7		18	18	
Drinking						
Yes	8	1	0.737	4	5	0.617
No	82	23		62	43	
Smoking						
Yes	49	12	0.698	36	25	0.795
No	41	12		30	23	
Body mass index						
≤25	73	20	1.00	55	38	0.571
>25	17	4		11	10	
T-staging						
T1	8	1	0.418	5	4	0.474
T2	27	5		22	10	
T3	29	7		18	18	
T4	26	11		21	16	
N-staging						
N0	10	0	0.240	8	2	0.219
N1	19	5		12	12	
N2	46	12		36	22	
N3	15	7		10	12	
Chemotherapy						
Yes	77	21	1.00	55	43	0.343
No	13	3		11	5	
Radiotherapy technique						
3D-CRT	40	8	0.327	29	19	0.642
IMRT	50	16		37	29	
Irradiation Dose (Gy)						
Mean±SD	42.37±5.09	43.16±4.43	0.493	29.48±10.16	30.92±10.38	0.460

Abbreviations: *IMRT* = intensity-modulated radiotherapy; *3D-CRT* = three-dimensional conformal radiation therapy, *SD* = Standard deviation. *Gy*=Gray.

## Discussion

To our knowledge, this study is the first investigation of genetic association between variants in *XRCC1* (*194Arg/Trp* and *399Arg/Gln*) and the risk of acute skin reaction and mucosa toxicity in NPC patients treated with RT. The other one studies involved tissue fibrosis and genotype analysis with much smaller sample size [10].

In our study, the frequencies of the variant alleles were 0.289 (*XRCC1-194Arg/Trp*) and 0.263 (*XRCC1-399Arg/Gln*), which were consistent with the previous literature data in Chinese subjects [16,17]. However, the allele frequencies were different from those previously reported in Caucasian populations (the variant alleles were 0.06-0.07 and 0.36-0.37) [18,19].

In this study, our data demonstrate that the risk of severe acute dermatitis up to grade 3 after radiotherapy could be increased by 2.65-fold in patients carrying with the *XRCC1 399Arg/Gln* genotype. There was no association between the *XRCC1 194Arg/Trp* genotype and skin reaction.

Although our observations have not been reported previously, they are strongly supported by information from relevant publications. The *XRCC1 399Arg/Gln* genotype has been reported to be associated with radiation sensitivity [8], mutagen sensitivity [9] and risk for lung cancer [20]. Furthermore, in a genotype-phenotype investigation, Au et al. [21] reported that the *XRCC1 399Arg/Gln* genotype was associated with reduced repair capacity for radiation-induced DNA damage but with normal repair of UV-light induced damage compared with the wild-type genotype. On the other hand, the *XRCC1 194Arg/Trp* genotype had repair activities similar to that of the wild-type. Therefore, our observations are likely to be real. Regarding the effects of the polymorphism of *XRCC1* on oral mucositis, there was no report about the association between *XRCC1* (*194Arg/Trp* and *399Arg/Gln*) genotypes and oral mucositis in the literature. However, polymorphisms in *XRCC3* were not associated with risk of oral mucositis [6].

Our results also showed that patients carrying with the *XRCC1* (*399Arg/Gln*) genotype trended to have higher incidence of grade 3 oral mucositis. The odds ratio was 2.11 (*p* = 0.065, *χ2* = 3.411). We did not find the relationship between polymorphism of *XRCC1* (*194Arg/Trp*) and oral mucositis. This further demonstrated that the *XRCC1* (*399Arg/Gln*) genotypes was linked to increased susceptibility of normal tissues to irradiation.

In the published literature, three reports were found which demonstrated the specific contribution of XRCC1 399Gln towards the expression of radiosensitivity. The first two reports by Hu et al. [8,22] showed that extensive radiation-induced cell cycle delay was associated with the variant gene in cells from normal individuals and from breast cancer patients. The third report [21] is from our co-author who demonstrated that the variant gene showed DNA repair of radiation-induced but not UV-induced DNA damage. However, whether the repair was normal or not was not investigated. Therefore, these three reports, in combination, revealed that the variant gene may have abnormal DNA repair function (e.g. mistakes in repair) which can cause radiosensitivity. In this context, the three reports validate our observation of radiation sensitivity towards dermatitis and mucositis in our patients.

## Conclusions

In summary, our study illustrates, for the first time, that patients with the *XRCC1* (*399Arg/Gln*) genotype were more likely to experience severe acute radiation-induced dermatitis and oral mucositis after radiation therapy. With further validation, the genotype information may be useful in determining personalized radiotherapy strategy.

## Competing interests

We declare no conflict of interest.

## Authors’ contribution

Each author had participated sufficient in the word. ZC and DL designed the research. HL, YY, CH and JC performed the genotyping analysis. CL and MZ collected the data and performed the statistical analysis. Finally, the manuscript was written by WWA. All authors read and approved the final manuscript.
